# Occupational Risk Factors for COPD: A Case-Control Study

**DOI:** 10.1371/journal.pone.0158719

**Published:** 2016-08-03

**Authors:** Marie Kraïm-Leleu, Francois-Xavier Lesage, Moustapha Drame, Francois Lebargy, Frédéric Deschamps

**Affiliations:** 1 Department of Occupational Health, Faculty of Medicine, University of Reims, Reims, France; 2 Department of Occupational Health, Faculty of Medicine, University of Montpellier, Montpellier, France; 3 Epsylon EA 4556, Department of Psychology. University of Montpellier, Montpellier, France; 4 Department of Public Health and Clinical Research, Faculty of Medicine, University of Reims, Reims, France; 5 Department of Respiratory Medicine, Faculty of Medicine, University of Reims, Reims, France; University of Pittsburgh, UNITED STATES

## Abstract

**Objectives:**

The aim of this research was to examine the occupational risk factors for Chronic Obstructive Pulmonary Diseases (COPD) in a range of occupations.

**Methods:**

Eleven occupations involving different types of exposure were observed in this multicenter case-control study. Controls and cases were matched for sex, age and smoking. Multiple logistic regression analyses were used to estimate odds ratios (ORs).

**Results:**

A total of 1,519 participants were initially recruited between September 2004 and September 2012. After matching, 547 pairs were obtained. The mean age was 56.3 +/- 10.4 years. Smelter workers were the only ones with an increased risk of COPD in this study (OR = 7.6, p < 0.0001, 95% CI [4.5, 12.9]). Physical activity was protective (OR = 0.7), while living in the city was a risk (OR = 1.6). The main used metals were cast iron, aluminum and alloys. Molds and cores were mainly made from sand and synthetic resins. Machine maintenance (65.2%), molding (49.6%), finishing (41.1%) and casting (41.0%) were the most common activities. Almost all workers (95.1%) cleaned the floors and machines with a brush or compressed air.

**Conclusions:**

This study demonstrates the importance of occupational factors in the genesis of COPD, especially among smelter workers. As with the fight against smoking-related disease, the removal or substitution of recognized hazardous agents is the best way of preventing the onset of COPD. This is why it is essential to continue research on its occupational risk factors.

## Introduction

Chronic obstructive pulmonary disease (COPD) is a slowly progressive respiratory disease with many potential causes. In 2004, the American Thoracic Society (ATS) and European Respiratory Society (ERS) defined COPD as “a preventable and treatable disease state characterized by airflow limitation that is not fully reversible” [1]. COPD is also associated with an abnormal inflammatory response of the lungs to noxious particles or gases, partly responsible for local emphysema lesions. Clinically, the symptoms characterizing COPD are phlegm, a productive cough lasting three months in two consecutive years, and effort dyspnea [1, 2].

In 2011, COPD was the sixth leading cause of death in developing countries, responsible for 4.9% of deaths [3], and it is expected to become the third leading cause worldwide by 2020 [4]. According to the World Health Organization (WHO), three million people die from COPD each year [5]. Although tobacco smoking is widely recognized as the dominant risk factor for COPD, other causes, such as environmental and occupational exposure need to be considered, too. In terms of costs, the annual burden of COPD is estimated at €38.7 billion in Europe, with 73% of costs related to inability to work, 12% to ambulatory care, 7.5% to hospitalization and 7.5% to medication [4]. Similarly, in the United States, the direct and indirect financial burden of COPD was put at $42.6 billion in 2007 [6]. In France, the mean direct annual cost per patient is estimated to be €4,366 [7].

The population attributable risk (PAR) for COPD associated with occupational exposure has been estimated at about 20% for smokers and 31% for nonsmokers [7]. In 2010, the ATS estimated that more than 20% of COPD cases are attributable to occupational exposure [8]. Epidemiological evidence also points to more than an additive effect between smoking and occupational exposure in the development of COPD [9–14]. Several studies worldwide have established that workplace exposure to vapors, gases, dust or fumes (VGDF) is partly responsible for the development of COPD [3, 15–17]. Moreover, a dose-dependent relationship has been found between exposure to dust, gas/fume and the presence of chronic pulmonary symptoms like cough and dyspnea [15]. The best documented sectors are mining, construction, foundry, welding, steel, textiles (especially cotton) and farming [17–19]. Construction workers exposed to inorganic dust have increased mortality due to COPD [20]. Lamprecht et al. demonstrated that farming should be regarded as a major risk factor for COPD [21]. Some findings suggest that prior occupational exposure may not only be associated with the development of COPD, but may also carry a risk of greater disease severity [22]. Furthermore the effect of occupational exposure is generally more marked in women [23]. Indoor air pollution has been shown to be a risk factor for COPD [17]. In addition, major urban pollution is thought to be responsible for an increase in the frequency of respiratory symptoms, breathlessness and decreased levels of pulmonary function [19, 24]. Other COPD risk factors include atopy, poor social conditions, malnutrition, a history of pulmonary tuberculosis, a history of repeated respiratory infections, passive smoking during childhood, and genetic predisposition [17, 25, 26]. We know that the most documented genetic risk factor is severe hereditary of alpha-1 antitrypsin deficiency [1, 24, 27]. However, occupational physicians have very few useful information at their fingertips to determine if a case of COPD is work related, either predominantly or partially [3].

COPD could be a work-related disease. Moreover, COPD could decreases the workers abilities too[9, 16, 28]. Blanc et al. found that occupational factors among people with COPD are associated with reduced quality of life, increased risk of restricted activity, and increased healthcare utilization [29]. According to Caillaud et al., 28.5% of COPD patients exposed to VGDF have to stop working definitively due to their respiratory problems [8]. COPD has also been found to have significant effects on absenteeism [3]. This economic argument is a further reason for studying the relationship between occupational exposure and the development of COPD regardless of age, sex and smoking. The goal of the present study was thus to identify those jobs and occupational factors most likely to contribute to the development of COPD. More specifically, we set out to study the relationship between COPD and a selection of occupations.

## Method

### Study design

A French multicenter case-control study has been implemented. Six respiratory medicine hospital departments and the occupational medicine department of Reims Champagne-Ardenne University took part in this research. The recruitment of the cases was done in departments of Champagne county hospitals, specialized to treat lung diseases. They have an assessment of their lung performances by spirometry. The criteria to be a case is the gold standard: the ratio FEV1 divided by FVC under 0.7, not reversible after salbutamol inhalation. The controls came from all departments of the same hospitals for a spirometry check, but they had not spirometric COPD criteria. During the recruitment phase, all individuals visiting one of the seven centers were invited to take part in the study. Participants were consecutively enrolled into the study. Participation was voluntary. The selected participants were aged 35–75 years. Controls and cases were frequency-matched for sex, age (+/-five years) and smoking (current, former, or nonsmokers).

Participants provided verbal informed consent to participate to this non interventional study. However, written consent form was not asked, considering this was a non interventional and anonymous study (the study began in 2004, the French law generalized the Ethic Committees for non-interventional studies since 2009). Authors received de-identified data from participants. No lung function measurements were carried out specifically for this study. These measurements were carried out in the usual medical examination frame. However, the occupational data were specifically collected for this study after the usual lung function measurement.

### Cases: participants with COPD

We based our selection of cases on the Global initiative for chronic Obstructive Lung Disease (GOLD)’s definition of Stage 1 COPD (ratio of post-bronchodilator Forced Expiratory Volume in one second [FEV_1_] to Forced Vital Capacity [FVC] < 70%). Individuals were excluded if they reported ever having had a diagnosis of asthma, cystic fibrosis, primary ciliary dyskinesia, bronchiectasis, primary or secondary pulmonary fibrosis, or emphysema with alpha-1-antitrypsin deficiency. Those with other chronic ear, nose and throat (ENT) diseases, including allergic rhinitis, were also excluded.

### Controls: participants without COPD

Individuals with a pre-bronchodilator FEV_1_/FVC ≥ 70% were selected as controls. Those with any significant respiratory or ENT diseases (see Cases: participants with COPD) were excluded.

### Variables and parameters collected

The variables required for matching were collected via an occupational physician-directed questionnaire. Smoking was estimated in pack-years, and smokers were divided into three categories: < 11 pack-years, 11–20 pack-years and > 20 pack-years. For the purposes of matching, participants were divided into two groups: smokers (current and former) and nonsmokers. The main questionnaire also contained a self-reported occupational history, allowing us to explore occupational exposure throughout the participants’ working lives. Participants were also asked about their possible exposure at home (e.g., carpentry, gardening, model building, and other leisure activities). Sport practice for more than an hour a week and place of residence (city or countryside) were also recorded.

### Measurement of occupational exposure

Another physician-directed questionnaire (a different one for each occupation) was administered to determine specific occupational exposure when a participant had worked for more than one year in one of the following sectors: farming, woodworking, textiles, rubber and plastics, tar/bitumen, tooling/machining, welding/brazing, glass, foundry, firefighting and pottery. Expert-directed self-assessments seem to yield better quality data than general self-report questionnaires or specific Job-Exposure Matrix (JEM) [30]. Inspired by the research conducted by Le Moual et al., our questionnaires were written in plain language so that they would be easily understood by employees [31]. Thus, most of the scientific terms and names of molecules were replaced by more common names. All questionnaires are available on request from the first author.

### Lung function measurements and definition of COPD

Lung function measurements were carried out by trained personnel in the seven centers. The spirometers were calibrated daily in accordance with the manufacturers’ instructions, and all equipment complied with European Commission standards.

COPD was defined as the ratio of post-bronchodilator FEV_1_ to FVC < 70% (COPD GOLD Stage 1 or above). The best of three FEV_1_/FVC results was chosen.

### Statistical analysis

The number of participants required in each group was calculated to be 560, for a minimum statistical power of 80%, an alpha risk of 5% and a minimum frequency of exposure in the control group of 5%. There was one control per case. After describing the sample, and the quantitative and qualitative variables, we used multivariate analysis to control for age, sex and smoking. Multiple univariate conditional regression models were used to estimate odds ratios (ORs) for the association between COPD and each of the 11 selected occupations. Multiple logistic regression analyses were then used to estimate the association between occupational exposure and COPD, taking other personal factors described above into account, such as leisure activities and place of residence. In a second step, we carried out descriptive studies of exposure factors for any occupation that had been recognized as a risk factor for COPD in our study. Additional details of the protocol are available from the first author. SAS 9.3 software (SAS Institute, Inc., Cary, NC), IBM SPSS Statistics version 2.1 and Microsoft Excel 2010 were used for the statistical analysis.

## Results

### Participants’ characteristics

A total of 1,519 individuals were initially recruited between September 2004 and September 2012 at the seven centers located throughout the Champagne-Ardenne region in France.

After matching for age, sex and smoking, 547 pairs were included in the analysis. The mean age of the 1,094 participants was 56.3 +/- 10.4 years (minimum = 27, maximum = 87, median = 55). There were 220 women (20.1%) and 874 men (79.9%). For smokers (*n* = 973/1,094), the mean number of pack-years was estimated at 38 +/- 22 (minimum = 1, maximum = 190, median = 34). Table 1 shows the distribution of cases and controls across the three smoking categories (<11, 11–20 and > 20 pack-years).

**Table 1:** Smoking categories.

smoking categories in pack-years	Casesn (%)	Controlsn(%)	Foundry workers*n(%)	Total n(%)
**<11**	31 (6.4)	39 (8.0)	11 (9.2)	70 (7.2)
**11–20**	64 (13.2)	63 (12.9)	25 (20.8)	127 (13.1)
**>20**	391 (80.4)	385 (79.1)	84 (70.0)	776 (79.8)
**Total**	486 (100)	487 (100)	120 (100)	973 (100)

n = 146 including 120 smokers

Approximately 80% (*n* = 776) of the smokers were in the smoking > 20 pack-years category. Regarding FEV_1_/FVC, the median was 61 (mean = 57.9 +/-10.6) for the case group and 78 (mean = 78.9 +/- 6.5) for the control group.

Completion rates for the eleven complementary questionnaires varied according to sector. The four most represented occupations were foundry (13.3%), machining/tooling (10.8%), farming (8.7%) and woodworking (7.3%). Regarding firefighting, we were only able to recruit one case matched with a control. Therefore, no statistical analyses could be performed for this occupation. Table 2 shows the distribution of cases and controls according to occupation and personal exposure, as well as bivariable relationship between baseline characteristics and risk of COPD. Due to a large amount of missing data, exposure durations could not be determined. By bivariable analyses, living in an urban area was associated with a significant risk of COPD (OR = 1.62, p < 0.0001, 95% CI [1.35, 1.94]). Lack of a leisure activity was also a risk factor (OR = 1.33, p = 0.01, 95% CI [1.06, 1.68]), whereas more than an hour of sport per week was a protective factor (OR = 0.65, p = 0.002, 95% CI [0.49, 0.86]). Despite the matching +/- five years, age was shown to be a risk factor for COPD. We therefore adjusted the results again for age in the multivariable analysis to reduce its effect.

**Table 2:** Sample description and occupational risk factors for COPD according to occupation and other personel factors (bivariable analysis).

	ORs	95%CI	*p*	Cases n (%)	Controls n (%)	Total n (%)
**Occupation**						
Farming	0.7	0.4–1.0	0.05	38 (6.9)	57 (10.4)	95 (8.7)
Woodworking	0.8	0.5–1.35	0.5	37 (6.8)	43 (7.9)	80 (7.3)
Textiles	1.7	1.0–3.0	0.06	37 (6.8)	23 (4.2)	60 (5.5)
Rubber and plastics	0.86	0.46–1.61	0.63	20 (3.7)	23 (4.2)	43 (3.9)
Pottery	0.33	0.11–1.03	0.06	4 (0.7)	12 (2.2)	16 (1.5)
Tar/bitumen	0.65	0.3–1.38	0.26	11 (2.0)	17 (3.1)	28 (2.5)
Firefighting	-	-	0.99	1 (0.2)	0 (0)	1 (0.1)
Tooling/machining	1.6	1.1–2.3	0.02	71 (13.0)	47 (8.6)	118 (10.8)
Foundry	6.9	4.1–11.4	<0.0001	123 (22.5)	23 (4.2)	146 (13.3)
Welding/brazing	0.65	0.38–1.1	0.11	25 (4.6)	37 (6.8)	62 (5.7)
Glassware	0.86	0.29–2.55	0.78	7 (1.3)	8 (1.5)	15 (1.4)
Others jobs	0.76	0.6–0.97	0.03	296 (54.1)	331 (60.5)	627 (57.3)
**Leisure activities and other factors**						
Carpentry	0.4	0.21–0.75	0.004	14 (2.6)	34 (6.2)	48 (4.4)
Gardening	0.89	0.69–1.14	0.34	178 (32.5)	193 (35.3)	371 (33.9)
Model building	1.14	0.41–3.15	0.8	8 (1.5)	7 (1.3)	15(1.4)
Other leisure activities	0.8	0.61–1.04	0.09	128 (23.4)	153 (28.0)	281 (25.7)
No leisure activity	1.33	1.06–1.68	0.01	257 (47.0)	215 (39.3)	472 (43.1)
Sport	0.65	0.49–0.86	0.002	123 (23.6)	170 (31.8)	296 (27.7)
Living in the countryside	0.82	0.64–1.06	0.13	217 (40.8)	195 (36.4)	412 (38.6)
Living in a city	1.62	1.35–1.94	<0.0001	315 (59.2)	341 (63.6)	656 (61.4)

By multivariable analysis, foundry was identified as a major risk factor for COPD (OR = 7.6, p < 0.0001, 95% CI [4.5, 12.9]). Tooling/machining and textiles were also found to be risk factors for COPD, but not significantly so. Most notably, farming and pottery were found to be protective factors. More than an hour of sport per week was protective against the disease (OR = 0.7, p < 0.05, 95% CI [0.57, 1.0]).

One hundred and forty-five foundry workers (123 cases and 22 controls) responded to the questionnaire on their occupational exposure. The results are reported in Table 3. The main metals used were cast iron, aluminum and alloys. Molds and cores were mainly made from sand and resins or synthetic resins (53.0%). The most common tasks were machine maintenance (65.2%), molding/coring (49.6%), trimming/finishing (41.1%) and casting (41.0%). Almost all the workers (95.1%) cleaned floors and machines with brooms or compressed air, but they were significantly (p = .049) more frequent in cases (96.7%) than in controls (85.7%). The molds and cores made from synthetic resins (78.5%) consisted primarily of phenol, formaldehyde and furan. The ovens were loaded with cast iron (48.0%), recycled scrap (63.9%) and coke (39.1%). Eighty one per cent of the deburring and finishing tasks performed by the affected employees involved shot blasting and 55.2% milling. The maintenance of machines and ovens primarily involved tar (67.6%), cement and refractory bricks (54.5%).

**Table 3:** Frequency of occupational exposure in smelters.

Foundry			
Exposures	Cases (*n* = 123) *n*/sample (%)	Controls (*n* = 22) *n*/sample (%)	Total (*n* = 145) *n*/sample (%)
Type of metal (molten, cast or molded) in the foundry			
Cast iron	62/122 (50.8)	18/23 (81.8)	80/144 (55.6)
Mild steel	6/119 (5.0)	7/20 (81.2)	13/139 (9.4)
Stainless steel and special steels	4/119 (3.3)	4/19 (21.0)	8/138 (5.8)
Aluminum (Al) and alloys	65/120 (54.2)	13/20 (65.0)	78/140 (55.7)
Copper, bronze and brass	4/120 (3.3)	12/20 (60.0)	16/140 (11.4)
Lead (Pb)	4/120 (3.3)	3/18 (16.7)	7/138 (5.1)
Other metals	1/113 (0.9)	5/12 (41.7)	6/125 (4.8)
Composition of molds and cores			
Green sand molds	31/118 (26.3)	6/17 (35.3)	37/135 (27.4)
Sand and resin or synthetic resin molds	60/117 (51.3)	11/17 (64.7)	71/134 (53.0)
Metal molds	13/116 (11.2)	6/15 (40.0)	19/131 (14.5)
Bricks and other refractory components	2/117 (1.7)	2/16 (12.5)	4/133 (3.0)
Other components	1/111 (0.9)	1/10 (10)	2/121 (1.6)
Tasks performed by participant or neighbor or in same workshop			
Sand preparation	23/120 (19.2)	14/20 (70.0)	37/140 (26.4)
Molding or coring	53/121 (43.8)	17/20 (85.0)	70/141 (49.6)
Loading the kiln	36/120 (30.0)	14/19 (73.7)	50/139 (36.0)
Casting	42/123 (34.1)	17/21 (80.9)	59/144 (41.0)
Knocking	22/121 (18.2)	13/19 (68.4)	35/140 (25.0)
Trimming and finishing	46/121 (38.0)	12/20 (60.0)	58/141 (41.1)
Furnace maintenance	33/121 (27.3)	10/19 (52.6)	43/140 (30.7)
Machine maintenance	83/121 (68.6)	7/17 (41.2)	90/138 (65.2)
Cleaning (furnaces or machines) with broom or blower	117/121 (96.7)	18/21 (85.7)	135/142 (95.1)
Other tasks	1/114 (0.9)	2/12 (16.7)	3/126 (2.4)
If sand preparation, molding or coring, contact with one or more of the following products			
	*n* = 76	*n* = 31	*n* = 107
Sand and molds heated	23/51 (45.1)	12/16 (75.0)	35/67 (52.2)
Sand	51/54 (94.4)	14/19 (73.7)	65/73 (94.5)
Black mineral/carbon black	28/53 (52.8)	7/15 (46.7)	35/68 (51.5)
Acids	4/48 (8.3)	6/14 (42.9)	10/62 (16.1)
Pitch/resin	4/52 (7.7)	1/13 (7.7)	5/65 (7.7)
Synthetic resins	46/51 (90.2)	5/14 (35.7)	51/65 (78.5)
If contact with synthetic resins, which ones?			
	*n* = 46	*n* = 5	*n* = 51
Phenol	46/46 (100)	2/3 (66.7)	48/49 (97.9)
Formalin	45/45 (100)	1/2 (50.0)	46/47 (97.9)
Polyurethanes	1/1 (100)	3/4 (75.0)	4/4 (100)
Furan	45/45 (100)	2/3 (66.7)	47/48 (97.9)
Epoxy	no data	2/3 (75.0)	2/3 (75.0)
Other synthetic resins	33/33 (100)	1/2 (50.0)	34/35 (97.1)
If loading furnaces			
	*n* = 36	*n* = 14	*n* = 50
Recycled scrap	10/36 (27.8)	6/11 (54.5)	16/47 (34.0)
Chromium	2/36 (5.6)	5/11 (45.5)	7/47 (14.9)
Nickel	2/36 (5.6)	5/11 (45.5)	7/47 (14.9)
Cadmium	2/36 (5.6)	0/9 (0.0)	2/45 (4.4)
Font	14/36 (38.9)	10/14 (71.4)	24/50 (48.0)
Other metals	26/36 (72.2)	4/11 (36.4)	30/47 (63.9)
Coal	3/36 (8.3)	3/9 (33.3)	6/45 (13.3)
Coke	12/36 (33.3)	6/10 (60.0)	18/46 (39.1)
Other products	0/31 (0.0)	2/10 (20.0)	2/41 (4.9)
If knocking			
	*n* = 22	*n* = 13	*n* = 35
Breaking molds by hand (hammer, pneumatic hammer)	12/17 (70.6)	11/13 (84.6)	23/30 (76.7)
Desanding of parts with compressed air	10/17 (58.8)	7/13 (53.8)	17/30 (56.7)
If trimming and finishing			
	*n* = 46	*n* = 12	*n* = 58
Sandblasting parts with compressed air	4/46 (8.7)	9/12 (75.0)	13/58 (22.4)
Milling	24/46 (52.2)	8/12 (66.7)	32/58 (55.2)
Sandblasting	5/46 (10.9)	7/12 (58.3)	12/58 (20.7)
Shotblasting	41/46 (89.1)	6/12 (50.0)	47/58 (81.0)
Gas cutting	3/45 (6.7)	3/11 (27.3)	6/56 (10.7)
Other finishing tasks	24/43 (55.8)	4/8 (50.0)	28/51 (54.9)
If maintenance of machines or ovens in person			
	*n* = 27	*n* = 14	*n* = 41
Using cement, refractory bricks	12/21 (57.1)	6/12 (50.0)	18/33 (54.5)
Using tar	21/21 (100)	2/13 (15.4)	23/34 (67.6)
Changing insulating plates (formwork ovens, skid plates, etc.)	11/21 (52.4)	5/13 (38.5)	16/34 (47.0)
Changing sealing joints of burners, thermal probes, resistors	10/21 (47.6)	4/13 (30.8)	14/34 (41.2)

## Discussion

This study demonstrates that occupational exposure to metal fume increases the risk of COPD, consistent with current literature [4, 13, 18, 32]. Lam et al. had previously reported an increased risk of COPD among workers exposed to high levels of coke oven emissions (OR = 5.80, 95% CI [3.13, 10.76]) [14]. Their study also showed that workers who loaded kilns were exposed to gas coke ovens. We also found that tooling/machining was a risk factor for the disease before adjustment for personal risk factors, as described in other studies and reviews [4, 18, 32, 33]. Whereas many studies have demonstrated a possible relationship between the inhalation of organic dust from cotton and pulmonary airflow limitation [4, 12, 18, 34], we failed to find a significant relationship between the textile industry and COPD.

Contrary to our expectations and to the literature [20], being a farmer was protective factor against the disease, albeit a statistically insignificant one (OR = 0.7, 95% CI [0.4, 1.0]). This result can be explained by the fact that our region is dominated by arable farming (cereals, vineyards), while the current data incriminate the endotoxins, dusts and ammonia associated with swine farming [13, 18, 33].

Among the individual risk factors, physical inactivity and lack of a leisure activity were significantly associated with an increased risk of COPD, as previously demonstrated by Blanc et al. [28]. More than one hour of sport per week was found to be protective, as it is for many other multifactorial diseases. However, one should be cautious in the interpretation of this finding. An odds ratio (OR) is a measure of association between two variables, but does not fulfill the required A.B. Hill criteria for causation. May the subjects without lung function diseases a more able to have a physical activity. However exercise is also known to improve the quality of life and prognosis of patients with COPD [35]. As described in the literature, outdoor air pollution is a risk factor for COPD, but causation is difficult to prove [16, 26, 36]. Moreover, Sunyer et al. have described the "urban factor" as a major risk factor for chronic bronchitis [37]. In the present study, living in a city was associated with a risk of COPD.

One strength of this study was that it evaluated the association between COPD and occupational exposure as assessed from lifetime occupational histories, whereas most previous case control studies have focused on the longest-held job or current job. Our sample included all age groups between 27 and 87 years. The inclusion of younger individuals may also explain why we found no increased risk for types of exposure known to be risk factors for COPD, insofar as participants had necessarily been exposed for shorter periods. To identify cases with COPD, we used the GOLD Stage 1 or above, which is an objective and reproducible definition. To optimize data collection and reduce recall bias, the questionnaires were completed with the help of an occupational physician or another trained professional. Questionnaires were also couched in terms that were familiar to the workers, in order to optimize their estimation of exposure. In addition, the questionnaires about the 11 selected occupations were based on the JEM model. We chose to conduct a case–control study because it offered us an opportunity to compare multiple types of exposure, including both occupational and personal exposure throughout the participants’ lifetime. Moreover, case-control studies are the most efficient study design for investigating the etiology of rare diseases or ones with an extremely low incidence [30].

The present study nonetheless had several limitations. Its multicenter nature may have introduced a selection bias and differences in how the data were collected. We limited this risk by training all the professionals who played a part in the study. Recall bias is presumably inevitable in any retrospective study, but in our study in particular, data on working life, the different occupations and, more especially, their respective durations, relied on the memory of our participants. Using a comprehensive questionnaire administered by an occupational physician partly minimized this bias. Our greatest challenge was to collect enough participants to create the 560 pairs needed to meet the study criteria. This is why we included individuals who were either younger or older than we had originally planned. Stricter matching criteria would have required a far longer recruitment phase (eight years instead of 16 months). Despite matching, age was associated with an increased risk of developing COPD. This can be explained by our use of a broad age interval when matching (+/- five years). Some occupations were underrepresented, such as firefighting, glassware and pottery. Significant results were found in the best represented occupations. The literature suggests that we would have had more significant results had there been more cases and controls in each sector. Some personal risk factors were not taken into account in our study, such as passive smoking in the home. Finally, like many studies of occupational risk factors, the healthy worker effect probably decreased the role of occupational exposure in the development of COPD.

This study once more demonstrates the importance of occupational factors in the genesis of COPD, especially among smelters. The importance of occupational factors in COPD and the latter’s expected increase over the coming years justify increased monitoring of exposed workers. Several studies have suggested that COPD patients with occupational exposure have a poorer prognosis and higher mortality than patients with no such exposure [13]. The role of the occupational physician is to preserve workers’ health. The removal of respiratory irritants and the substitution of nontoxic agents for toxic ones are the best ways of eliminating work-related COPD risk, as they are in the fight against active and passive smoking. Moreover, clinicians must be aware of these risks and ask all new patients with COPD about their occupational history in order to improve their prognosis by stopping all exposure, trigger the payment of work-related insurance compensation, and participate in public health surveillance. Educating employees about the dangers of their occupational exposure, the collective and individual forms of protection required, and also the first signs of COPD, are also part of prevention. To reduce the burden of COPD worldwide, it is essential to continue research on occupational risk factors. New case-control studies within specific occupations are needed to deepen our understanding and complement the work accomplished in the present study.

## Conclusions

The present study uncovered a high prevalence of COPD in smelters. A major global health problem, COPD is a chronic, progressive and disabling disease with multiple risk factors. Occupational exposure is responsible for 20% of all cases of COPD [12], and worsens the prognosis in cases of continued exposure. Mortality, plus the direct and indirect costs of its care in industrialized countries, largely justifies research to increase our knowledge of these occupational risks in order to prevent them more effectively.
